# Multimodal Performance Prediction in Online Learning Using Bi-LSTM and Mixture of Experts

**DOI:** 10.3390/s26216874

**Published:** 2026-10-30

**Authors:** Qikai Lin, Jingyu Liu, Yunhong Ding, Bo Qi

**Affiliations:** 1 School of Computer Science and Information Engineering, Harbin Normal University, Harbin 150001, China; 2024300716@stu.hrbnu.edu.cn; 2 School of Astronautics, Harbin Institute of Technology, Harbin 150001, China; 22b918100@stu.hit.edu.cn

**Keywords:** camera-based sensing, classroom affective–behavioral analysis, temporal affective modeling, academic performance prediction, multisource data fusion, multimodal learning analytics

## Abstract

Educational records alone may not capture changes in students’ classroom affective states. This study proposes Bi-LSTM-SCPM, a student comprehensive performance model (SCPM) integrating bidirectional long short-term memory (Bi-LSTM) with mixture of experts (MoE) regression. The dataset comprised 14,645 facial images from 71 students across three stages of 12-week computer-assisted translation courses. ResNet50 achieved 93.8% accuracy in positive–negative facial state classification. Bi-LSTM integrated stage scores derived from instructor-designated key periods and Bayesian smoothing into a student-level affective score (ZHCJ). ZHCJ was combined with educational information and prerequisite course performance to predict final grades. Across 30 matched student-level splits, MoE achieved an R^2^ of 0.943 ± 0.002 and an MAE of 1.144 ± 0.128 points, compared with 0.927 ± 0.002 and 1.274 ± 0.149 points for XGBoost. Both differences remained significant after Holm adjustment (*p* < 0.001). ZHCJ also outperformed summary statistics and direct stage score inputs under both predictors. The setting ϖ = 2 and k = 20 performed best within the nine-setting grid. These findings support the predictive value of integrating temporal affective representations and educational information within the study cohort.

## 1. Introduction

### 1.1. Research Background

Academic performance prediction increasingly combines structured educational records with data on learning processes, affective states and online interactions. In online classrooms, where direct feedback can be limited, facial expressions provide an additional source of information for assessing learning engagement [1]. Aly applied deep learning to facial expression recognition for real-time tracking of students’ online learning engagement [2]. Recent work has also introduced Vision Transformer (ViT) models with hybrid local attention for facial expression recognition [3]. Beyond recognizing expressions in individual images, temporal modeling can capture changes in facial features across classroom videos. Chen et al. proposed a spatiotemporal residual attention network (STRAN) for student expression recognition in classroom videos, reporting an accuracy of 78.19% [4]. Maddu and Murugappan developed a hybrid model combining ResNet, AAM, SLBT and GBP features, achieving 95% accuracy in real-time engagement detection in virtual learning environments [5]. These studies illustrate how facial expression data can support the assessment of learning states across educational settings.

This study combines context-aware scoring, Bayesian smoothing and Bi-LSTM temporal modeling to predict academic performance. Facial expression sequences are aligned with instructor-designated key periods to derive temporal affective features, which are then integrated with students’ demographic and academic information.

### 1.2. Related Work

#### 1.2.1. Learning Records and Performance Prediction

Early studies of academic performance prediction relied mainly on structured behavioral data and conventional machine learning algorithms. Liu et al. compared conventional models with long short-term memory (LSTM) networks using clickstream data from the Open University Learning Analytics Dataset (OULAD). LSTM achieved better predictive performance, demonstrating the value of preserving temporal information in learning behavior [6]. Deep neural networks have also been used to predict performance early from sequences of online learning activities [7]. These approaches capture behavioral patterns over time but do not explicitly model students’ affective states. Beyond behavioral logs, Cui developed a natural language processing (NLP) system that assessed English writing using lexical complexity, grammatical accuracy and discourse features [8]. Its focus was textual quality rather than affective changes during writing. More recent studies have extended prediction to multidimensional time-series data [9] and combined video, text and logs for engagement assessment [10]. This progression motivates combining temporal affective features with structured educational information for academic performance prediction.

#### 1.2.2. Multimodal and Temporal Modeling

Multimodal learning analytics combines images, text and physiological signals to examine affective changes and learning behavior. Zhai et al. combined facial expressions and head poses using a convolutional neural network (CNN) with attention, improving affect recognition accuracy over either source alone [11]. Hernández-García et al. analyzed learning management system (LMS) interactions across multiple courses using a learning cycle framework. Their findings supported the predictive relevance of LMS activity while showing that its association with academic performance varied between online and blended teaching settings [12]. These log-based analyses inform performance prediction but do not directly capture continuous changes in students’ affective states.

Recent work has explored multimodal large language models and graph-based methods for analyzing visual and educational data. Yu et al. evaluated Gemini for automated emotion recognition using classroom videos and static images [13]. Graph-based methods offer another way to represent relationships in visual data. Cheng et al. reviewed graph convolutional networks (GCNs) for image restoration [14]. In an educational application, Li et al. combined a 3D Haar semi-tight framelet transform with graph structure learning for student engagement prediction, modeling relationships across modalities [15].

For emotion recognition from electroencephalography (EEG) signals, Yao et al. developed EEG ST-TCNN, a hybrid Transformer–CNN model. Two parallel Transformer encoders extract spatial and temporal features, which a CNN then combines for classification. The model achieved 96.67% accuracy on SEED and 95.73–96.95% across three classification tasks on DEAP [16]. In educational settings, Alarefah et al. combined a pre-trained ViT for spatial feature extraction with an LSTM for temporal modeling in a prototypical few-shot learning framework. They evaluated the model on EngageNet for engagement recognition with limited labeled examples [17].

Outside education, Yang et al. proposed Surgformer for surgical phase recognition. The model combines divided spatiotemporal attention with hierarchical temporal attention (HTA) to capture local and global information from sparsely sampled video frames and was evaluated on two surgical video benchmarks [18]. Chen et al. developed a modified Transformer that used robotic-arm kinematics for surgical gesture recognition, gesture prediction and end-effector trajectory prediction. It achieved up to 94.4% gesture recognition accuracy on JIGSAWS [19].

This study constructs the Temporal Multimodal Student Assessment (TeMSA) dataset by combining facial expression sequences, structured educational information and prerequisite course performance. A bidirectional long short-term memory (Bi-LSTM) network integrates affective scores from the early, middle and late course stages to assess their joint predictive contribution to final grades. The resulting student-level representation is combined with educational predictors for mixture of experts (MoE) regression.

### 1.3. Research Objectives and Contributions

This study has the following objectives:

1. Develop a context-aware scoring method that assigns larger positive contributions and negative deductions during instructor-designated key periods and use Bayesian smoothing to estimate stage scores.
2. Use Bi-LSTM to integrate affective scores from the early, middle and late course stages into a student-level temporal affective representation.
3. Construct the TeMSA dataset by combining facial expression sequences, structured educational information and prerequisite course performance to evaluate the added predictive value of temporal affective features.
4. Compare individual regression models with MoE for final grade prediction using the same student-level training and test splits.
5. Use a gating network to combine random forest (RF), XGBoost, CatBoost and ridge regression in an MoE model. Analyze the expert weights to describe how model contributions vary across students.

## 2. Materials and Methods

### 2.1. Problem Formulation

#### 2.1.1. Research Questions

Research has linked academic emotions to self-regulated learning and achievement [20] and emotional intelligence to academic performance [21]. This study examines whether affective features derived from classroom facial expressions improve final grade prediction when combined with students’ demographic and academic information.

This study treats final examination score prediction as a regression task. Predictors include demographic information (e.g., gender, political affiliation and place of origin), academic background (e.g., major and prerequisite course performance) and affective features derived from classroom facial expressions. Bi-LSTM integrates scores from the early, middle and late course stages into the affective score ZHCJ. Direct stage scores and alternative affective representations are evaluated in separate model configurations.

This study addresses the following research questions:

RQ1: How well do affective scores predict final grades when used separately for the early, middle and late course stages or integrated across stages, with greater weight given to instructor-designated key periods?

RQ2: Does adding classroom affective features to students’ demographic and academic information, including prerequisite course performance, significantly improve final grade prediction?

RQ3: Does the Bi-LSTM-derived score ZHCJ significantly improve final grade prediction compared with summary statistics and direct inputs of the three stage scores?

#### 2.1.2. Mathematical Representation

Let the set of students be $S=\{s_{1},s_{2},\ldots ,s_{n}\}$. For any student $s_{i}\in S$:

Basic Information Feature Vector: $X_{i1}=[x_{i11},x_{i12},\ldots ,x_{i1m_{1}}]$, where $x_{i11}$ denotes the gender, $x_{i12}$ denotes the political status (e.g., general public, Communist Youth League member, Party member), $x_{i13}$ denotes ethnicity and $x_{i1j}$ represents features related to the student’s place of origin.

Academic Background Feature Vector: $X_{i2}=[x_{i21},x_{i22},\ldots ,x_{i2m_{2}}]$, where $x_{i21}$ represents the usual course performance, and $x_{i2j}$ is the classroom emotion scores for the early, middle and late stages of the semester, respectively.

Comprehensive emotion fluctuation index is $x_{i3}$.

The input feature vectors $X_{i}=[X_{i1},X_{i2},x_{i3}]$, where their dimensions are $m=m_{1}+m_{2}+1$.

Let the final exam score be $z_{i}$. The regression prediction can be expressed as:

(1) $$z_{i}=f(X_{i})+\epsilon_{i}$$

where f is the prediction model function, $\epsilon_{i}$ represents the prediction error and the goal is to ensure the predicted value ${\hat{z}}_{i}=f(X_{i})$ as close as possible to the true final exam score $z_{i}$ through training data to form the model parameters f.

### 2.2. Dataset

#### 2.2.1. Study Setting and Data Collection

The dataset comprises student classroom facial expression images (SCFEIs), educational information (EI) and emotion quantification scores (EQC). Data were collected in computer-assisted translation courses attended by undergraduate and postgraduate students majoring in English translation at the School of Western Language and Culture of a university in Harbin.

SCFEI contains facial images captured from multiple camera views during online classes and annotated with facial state labels. EI includes demographic attributes such as gender, ethnicity and place of origin. EQC comprises numerical scores derived from facial state recognition outputs, with greater weight given to observations during instructor-designated key periods.

Recordings were collected via online video conferencing during the early (Weeks 1–4), middle (Weeks 5–8) and late (Weeks 9–12) stages of the 12-week courses. Recording sessions followed standardized classroom procedures and predefined instructional plans.

Recordings included students’ faces and instructors’ shared screen content. Instructors identified key instructional periods for subsequent scoring. Before data collection, participants provided written informed consent after receiving information about the research purpose, collection procedures, intended data use and privacy safeguards. Course instructors were not part of the research team and had no access to the research recordings, facial images, individual affective scores or prediction results. Table 1 summarizes the video data.

#### 2.2.2. Facial Emotions of Students in Classroom

Facial expressions can provide nonverbal cues to students’ classroom affective states. Educational research associates enjoyment and engaged concentration with productive learning, while persistent boredom is linked to poorer engagement and outcomes. These relationships vary with the affective state, its duration and the learning context [22,23,24]. Temporal modeling can account for dependencies between successive observations. Pei et al. combined recurrent neural networks with Bayesian filtering for continuous affect recognition from facial image sequences [25].

Positive–negative labels were used for classroom state classification. Facial action unit (AU) descriptors were extracted programmatically from sampled images to characterize observable facial movements. Related work includes multimodal ensemble methods for micro-expression recognition [26] and BP4D+, a corpus annotated using the Facial Action Coding System (FACS) [27]. Micro-expression spotting has also been studied using multimodal methods [28] and optical flow with recurrent neural networks [29]. AU extraction alone does not demonstrate detection of brief micro-expression events. Subsequent temporal modeling integrates affective scores from the early, middle and late course stages.

Six observable classroom states defined the binary labels (Table 2). Focused and Pleasure were labeled positive; Head-down, Phone-using, Head-turning and Drowsiness were labeled negative. To assess labeling reproducibility, 600 images were randomly sampled without replacement from the designated test set, covering 21 students, and independently labeled by two annotators. Agreement was calculated from the independent label sets. This analysis assessed the reproducibility of the operational labels, rather than their validity as direct measures of internal emotions.

**Figure 1:**
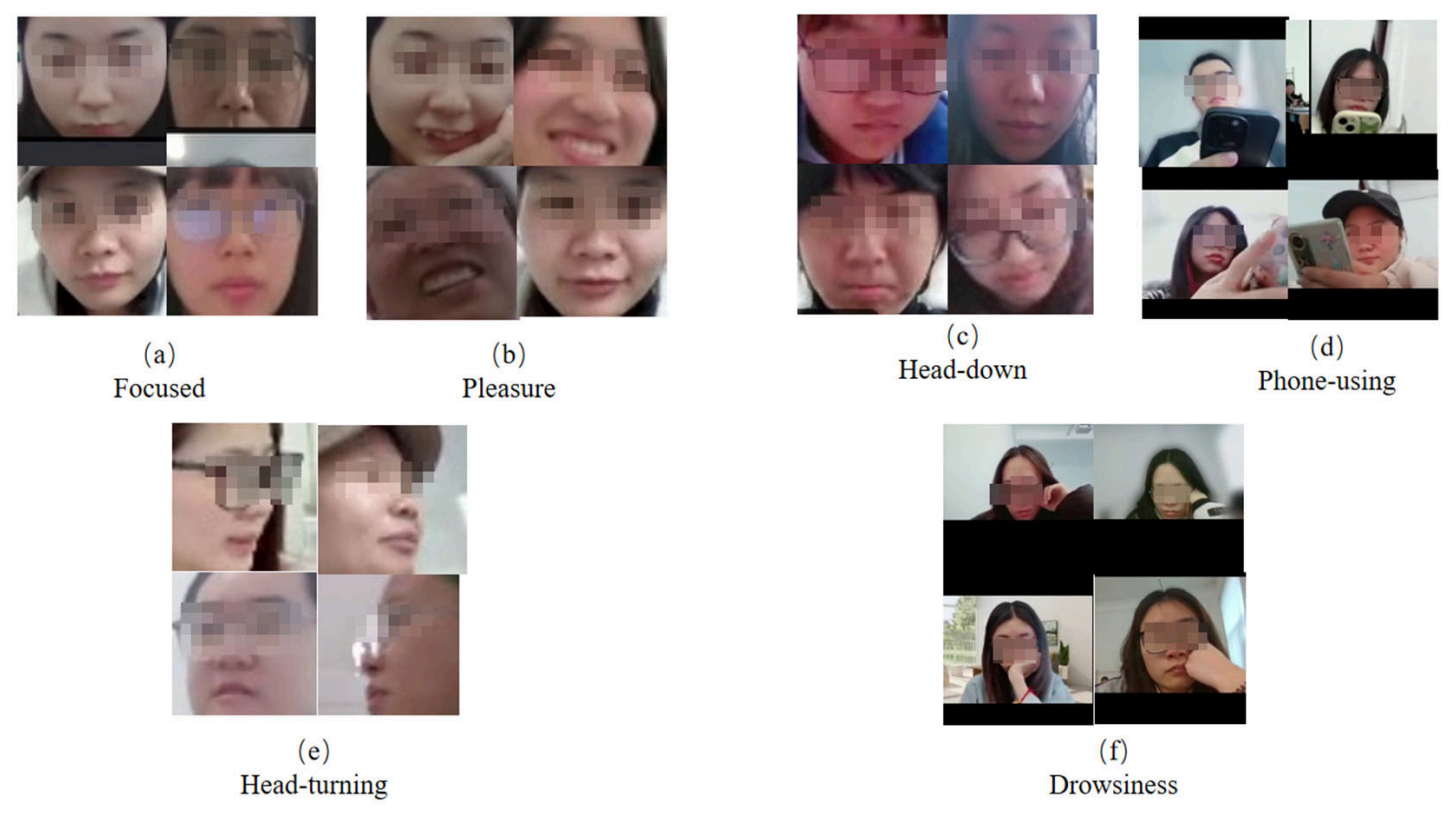
Examples of the six classroom states.

Figure 1 shows examples of the six classroom states, and Table 2 summarizes their operational definitions in the TeMSA dataset.

#### 2.2.3. Data Preprocessing

(1) Educational information

Sixteen student records with substantial missing feature data were excluded from analysis. Missing values arose from circumstances such as absence from class or course exemption.

Feature encoding and normalization were performed after the data were split by student. The same encoding scheme was applied to the training and test sets. Normalization parameters were estimated from the training set alone and used to transform both sets.

The EI dataset comprised demographic information and academic records for 71 students. Table 3 summarizes the six predictor variables and the final examination score used as the prediction target.

(2) Classroom facial images

Images from multi-camera recordings were manually checked within each student’s recording session. Duplicate records of the same student at the same sampling time were removed before image classification and stage score calculation to avoid repeated counting.

Python scripts extracted frames every 20 s from classroom videos across the three course stages. The interval was chosen to balance processing and manual annotation costs with regularly spaced observations; alternative intervals were not compared experimentally. Positive–negative image classifications were aggregated into stage scores for final grade prediction.

This approach characterizes classroom states at sampled time points for aggregation across course stages. Brief facial events may occur entirely between sampled frames and remain undetected. Frame-level AU descriptors cannot recover unobserved movements, so the procedure does not provide continuous coverage of micro-expression dynamics [29]. The results do not establish that the 20 s interval is optimal or captures every affective event.

Image preprocessing included resolution checks, denoising, object detection and restoration and illumination normalization [30,31]. Images with low resolution, excessive noise, occlusion or insufficient lighting were excluded. After student-level partitioning, the same preprocessing rules were applied separately to the training and test sets. Class labels, academic outcomes and test set results were not used to determine preprocessing settings. Model training and evaluation followed the protocol in Section 2.2.4. Previous work has examined preprocessing for object recognition [32] and denoising for image restoration [33]. These steps yielded the usable classroom image dataset.

Manual inspection identified duplicate multi-camera records within each student’s recording session. Duplicate records referred to images of the same student at the same sampling time. Images from different time points were not removed solely because expressions or postures were similar. Removing 5812 duplicates from 20,457 images left 14,645 images for annotation, classification and stage score calculation.

Images were assigned positive–negative labels according to Section 2.2.2 and stored in the corresponding class directories to form the FaceImage dataset.

#### 2.2.4. Dataset Partition and Experimental Protocol

To prevent student-level leakage, the 71 students were randomly assigned to training and test sets in an approximately 7:3 ratio. All facial images, affective scores, demographic information and academic records from each student remained in the same set. The student was the independent sampling unit; the 14,645 images were repeated observations within 71 students. Each student contributed one three-stage score sequence for temporal modeling.

Models were fitted on the training set and evaluated on the test set. Thirty repeated student-level splits supplemented the initial held-out evaluation to assess sensitivity to sample allocation (Section 2.4.1). Each split maintained student-level separation between the training and test sets.

### 2.3. Bi-LSTM-SCPM Framework

This study proposes Bi-LSTM-SCPM (student comprehensive performance model), which combines Bi-LSTM-based affective representation learning with MoE regression to predict final course grades in online learning. Bi-LSTM integrates the three stage scores into ZHCJ, a student-level affective representation. ZHCJ is combined with structured educational information and prerequisite course performance. MoE combines predictions from random forest (RF), XGBoost, CatBoost and ridge regression using student-specific gating weights.

The Bi-LSTM-SCPM framework consists of five main modules:

#### 2.3.1. Facial State Classification and Bayesian Scoring

**Figure 2:**
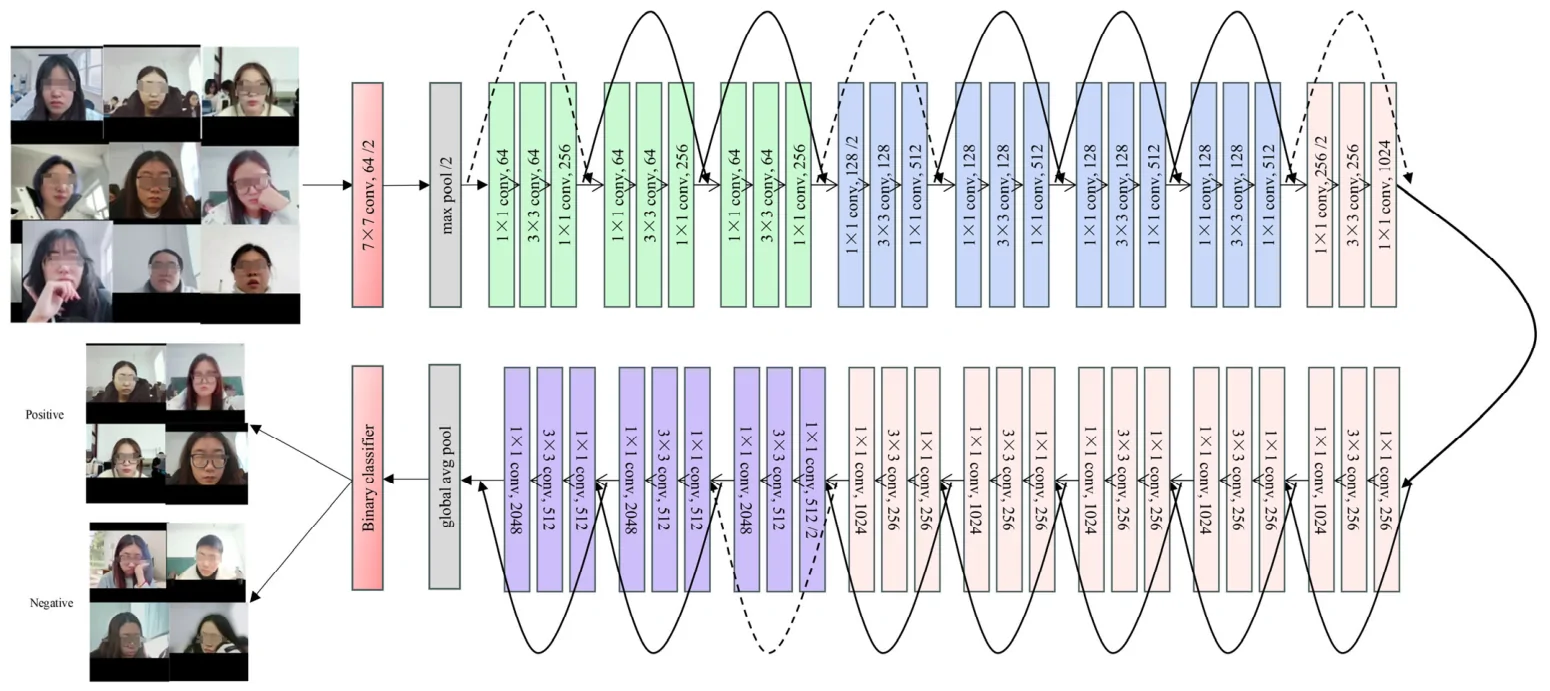
ResNet50-based facial state classification architecture. Arrows indicate the direction of feature propagation. Arched shortcut arrows denote residual connections, with dashed shortcuts indicating transitions where feature-map dimensions change.

This study used ResNet50, Vision Transformer (ViT) and a hybrid model for positive–negative classification of classroom facial images. ResNet50 extracts facial features through convolutional layers with residual connections. Lin and Wang reported accuracies of 73% and 99.57% on FER2013 and JAFFE, respectively, using an adaptive semi-supervised model based on improved ResNet50 [34]. Their model outperformed the CNN baselines included in the comparison. Figure 2 shows the ResNet50-based classifier used in this study.

ViT uses self-attention to model global relationships within facial images for facial state classification. Tian et al. combined ViT with hybrid local attention for facial expression recognition [3]. Transformer-based architectures have also been applied to vehicle detection [35]. Hybrid architectures combine convolutional feature extraction with global attention; Li et al. incorporated multi-scale feature integration and patch dropping for facial expression recognition [36].

All three classifiers used 224 × 224-pixel RGB images, a batch size of 128 and categorical cross-entropy loss. The classifiers were trained for 100 epochs at fixed learning rates, and their accuracy curves showed a typical convergence trend. Model-specific optimizer settings, learning rates and classification head dropout rates are summarized in Table 4.

Training augmentation included rotations within ±15°, translations of up to 10% of image width or height, zooming within 10%, horizontal flipping and brightness factors of 0.900–1.100. Random augmentation was not applied to validation or test images. Configurations were fixed before test evaluation, without using test results to select hyperparameters or training duration.

This study uses a dynamic scoring strategy that combines key period weighting with Bayesian smoothing. Scores were calculated from deduplicated images so that each student’s observation at a given sampling time was counted once. Deep learning classifiers assigned positive–negative labels to the sampled facial images. Observations from teacher-designated key instructional periods received greater weight, as defined in Equations (2) and (3).

(2) $$P=P_{normal}+\varpi \cdot P_{focus}$$

(3) $$N=N_{normal}+\varpi \cdot N_{focus}$$

where P and N denote the weighted counts of positive and negative states, respectively. $P_{normal}$ and $N_{normal}$ are the corresponding counts during non-key instructional periods. $P_{focus}$ and $N_{focus}$ are the corresponding counts during teacher-designated key instructional periods. The coefficient ϖ controls the weight assigned to observations from these key periods.

Bayesian approaches have been used to represent uncertainty in emotion prediction [37]. In this study, Bayesian smoothing was applied to stabilize stage score estimates for students with few usable images. Pseudo-counts based on a prior expectation were combined with the weighted positive and negative counts to obtain the smoothed proportion in Equation (4).

(4) $$r_{adj}=\frac{b+k\cdot r_{0}}{b+l+k}$$

where $r_{adj}$ denotes the smoothed positive-state proportion used for subsequent modeling. The quantities b and l represent the positive and negative counts for a given student, respectively. The prior expectation $r_{0}$ was estimated as the mean positive-state proportion in the training data. The parameter (k) controls the strength of the prior through pseudo-counts.

The parameters ϖ and k have distinct roles: ϖ weights observations from teacher-designated key instructional periods, whereas k controls smoothing strength. In the main analysis, ϖ = 2 and k = 20, so key period observations received twice the weight of non-key observations. The value of k refers to pseudo-count strength on the scale used in the scoring formula and does not denote a student count. Within each student-level split, $r_{0}$ was estimated using training students only. Sensitivity to both parameters was examined as described in Section 2.4.3.

When k is large relative to b+l, the smoothed estimate $r_{adj}$ is closer to the prior expectation $r_{0}$. As b+l increases, the observed data have greater influence, and $r_{adj}$ approaches the empirical proportion $\frac{b}{b+l}$.

Stage scores express the smoothed positive-state proportion on a 0–100 scale. Higher scores indicate a greater weighted proportion of observations classified as positive. The smoothing procedure provides a point estimate but does not itself yield a confidence interval or quantify uncertainty across the prediction pipeline. A score near 50 does not necessarily indicate a psychologically neutral state.

#### 2.3.2. Temporal Modeling and Feature Extraction

**Figure 3:**
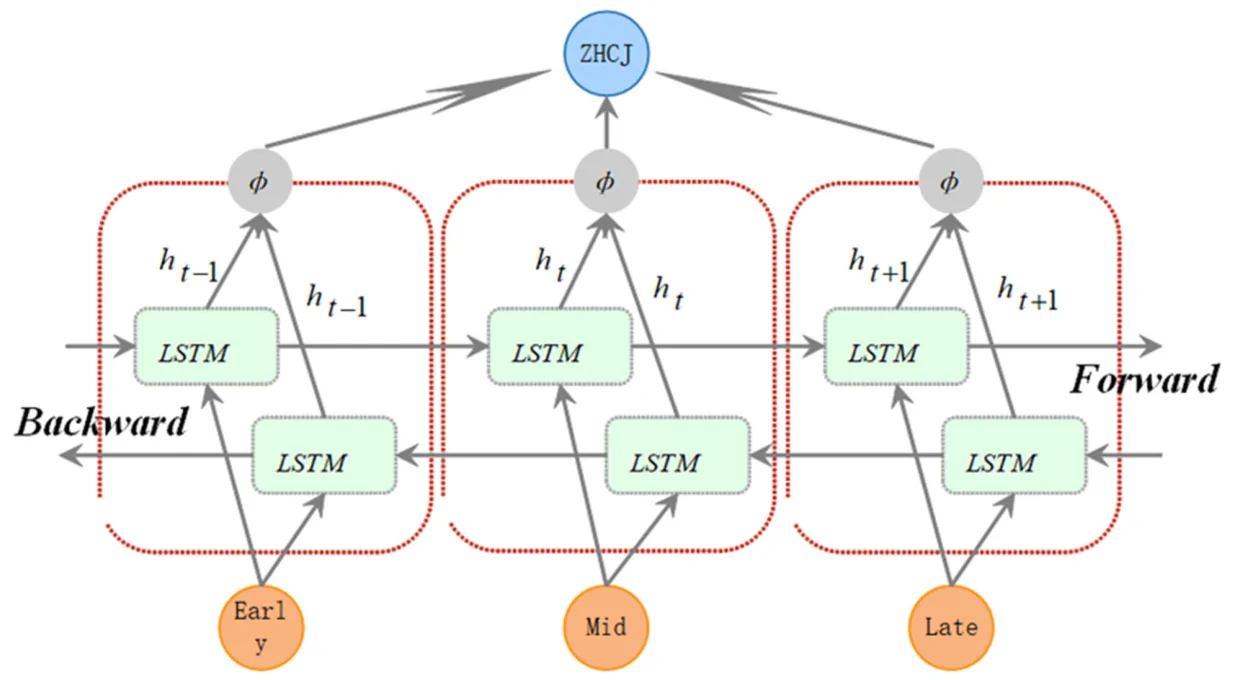
Bi-LSTM architecture for integrating three-stage affective scores. Horizontal arrows indicate the two directions of sequence processing, while the other arrows indicate information flow from the stage inputs through the LSTM units and fusion nodes (ϕ) to ZHCJ.

In this study, Bi-LSTM integrated affective scores from the early, middle and late course stages into a student-level score, ZHCJ. The forward and backward branches process the three stages in opposite directions to incorporate bidirectional context. Because the sequence contains only three time steps, this design does not demonstrate an ability to model long-range temporal dependencies. ZHCJ was compared with summary statistics and direct inputs of the three stage scores to assess its added predictive value under matched evaluation conditions. Figure 3 illustrates the Bi-LSTM architecture used to integrate these stage scores. Because this representation requires all three stages, prediction was evaluated after late-stage observations became available.

LSTM-based models have been applied to both student performance prediction and emotion recognition. Qin et al. proposed MFO-ATTENTION-LSTM, combining moth-flame optimization with an attention-based LSTM to improve in-class performance prediction from learning logs [38]. Nanavaty and Khunteta used LSTM with attention to predict online learner performance from temporal patterns in assessment and engagement [39]. For speech emotion recognition, Li et al. developed Graph-LSTM, which combines an LSTM aggregator with weighted graph pooling [40].

#### 2.3.3. Prerequisite Course Modeling and Feature Construction

This study incorporated grades from two prerequisite courses closely related to computer-assisted translation to account for differences in students’ prior academic preparation. The undergraduate courses were Translation Theories and Practice I and Literary Reading and Translation I. The postgraduate courses were Introduction to Translation Theories and Intermediate Translation. Using these grades as separate predictors may not explicitly capture inter-course relationships or their relative contributions to performance in the target course. This study therefore adopted knowledge state mapping (KSM) to construct a standardized fused feature representing students’ overall mastery of relevant prerequisite knowledge.

Each prerequisite course grade was first standardized and then mapped to a mastery score using Equation (5).

(5) $${\hat{K}}_{i}=\sigma \left(\frac{q_{i}-\mu_{i}}{\sigma_{i}}\right)$$

where $\mu_{i}$ and $\sigma_{i}$ denote the mean and standard deviation of grades for prerequisite course i, respectively. The sigmoid function σ(⋅) maps standardized grades to the 0–1 range to represent mastery of the corresponding prerequisite knowledge.

Fusion weights $\lambda_{i}$ were assigned according to each prerequisite course’s contribution to computer-assisted translation, as specified in the syllabus. The two mastery scores were then combined into a weighted feature using Equation (6).

(6) $$K_{\mathrm{fused}}=\lambda_{1}\cdot {\hat{K}}_{1}+\lambda_{2}\cdot {\hat{K}}_{2}$$

Based on instructors’ expert assessments, this study set the undergraduate weights to $\lambda_{1}$ = 0.6 and $\lambda_{2}$ = 0.4. For postgraduate students, the corresponding weights were $\lambda_{1}$ = 0.5 and $\lambda_{2}$ = 0.5. The fused feature summarizes prerequisite knowledge relevant to the target course and was used as a structured predictor alongside the temporal affective features.

This feature construction draws on knowledge tracing and learning state modeling, which represent learners’ knowledge mastery to support performance prediction. Zhang et al. proposed dynamic key value memory networks that store knowledge concepts and update corresponding mastery levels as learning progresses [41]. In the present study, prerequisite course grades serve as indicators of prior knowledge. The resulting fused feature provides a structured summary of students’ academic preparation for the target course.

#### 2.3.4. Multi-Source Feature Fusion

The TeMSA dataset combines classroom affective information with structured educational records. Available affective variables include early-, middle- and late-stage scores and the Bi-LSTM-derived integrated score, ZHCJ. The input combinations used in each comparison are defined in Section 2.4.1.

The original field names were retained: XBMC denotes gender, MZMC ethnicity and ZZMMMC political affiliation. JTDZ denotes the recorded residential address, and PSCJ denotes the coursework score. Ethnicity remained an input variable, although the cohort contained only one ethnic category, precluding ethnicity-based subgroup analysis. The prerequisite course performance feature was constructed as described in Section 2.3.3. ZJCJ, the final course grade, was the prediction target and was excluded from the predictors. Categorical encoding and numerical scaling were fitted using only training data and then applied to the corresponding test data.

Previous studies support integrating complementary information for student performance prediction. Chango et al. combined attribute selection and ensemble learning to improve performance prediction using multimodal data from an intelligent tutoring system [42]. Hernández-García et al. examined the relationship between learning management system (LMS) interactions and academic performance using a learning cycle approach [12].

#### 2.3.5. Regression-Based Prediction and Performance Evaluation

1. Machine Learning Models

This study used CatBoost, random forest (RF), XGBoost and ridge regression to predict final course grades from the multimodal feature vectors.

CatBoost is a gradient-boosted decision tree algorithm with support for categorical features. Its ordered boosting and categorical encoding strategies are designed to reduce prediction bias and limit overfitting. Abed applied CatBoost in a hybrid model for demand forecasting [43].

RF averages the predictions of multiple decision trees to model nonlinear relationships and interactions among predictors. Combining trees can reduce prediction variance and improve model stability. RF regression has also been used to predict students’ academic achievement and fuzzy grades [44].

XGBoost (eXtreme Gradient Boosting) uses second-order gradient information and regularization to fit boosted decision trees efficiently. Regularization helps control model complexity and limit the risk of overfitting. Liang et al. used XGBoost for risk prediction from structured clinical data [45].

Ridge regression adds an $L_{2}$ penalty to the least-squares objective, shrinking coefficients and improving numerical stability when predictors are highly correlated. By controlling coefficient magnitudes, it can reduce estimation variance and limit model complexity in multicollinear feature spaces. Akhtar and Alharthi examined condition-adjusted shrinkage parameters and reported improved estimation performance in simulations and a real-data application with severe multicollinearity [46].

2. Performance Evaluation

Regression performance was evaluated using mean squared error (MSE), mean absolute error (MAE) and the coefficient of determination (R^2^) [47]. These metrics describe squared prediction error, absolute prediction error and performance relative to a mean-based reference, respectively. MAE and MSE are expressed in grade points and squared grade points, whereas R^2^ is dimensionless. Their definitions are provided below.

(1) Mean Squared Error (MSE): MSE measures the average of the squared differences between predicted values and ground truth values. By squaring the errors, it assigns greater penalties to larger deviations, making it especially suitable for regression tasks in which large prediction errors are of particular concern.

(7) $$\mathrm{MSE}=\frac{1}{n}\sum_{i=1}^{n}{\left(y_{i}-{\hat{y}}_{i}\right)}^{2}$$

where $y_{i}$ and ${\hat{y}}_{i}$ denote the observed and predicted grades for student i, respectively, and n is the number of evaluated students.

(2) Mean Absolute Error (MAE): MAE computes the average of the absolute differences between the predicted values and the ground truth values, reflecting the magnitude of the average prediction error and offering intuitive interpretability.

(8) $$\mathrm{MAE}=\frac{1}{n}\sum_{i=1}^{n}\left|y_{i}-{\hat{y}}_{i}\right|$$

(3) Coefficient of Determination (R^2^): R^2^ compares the model’s squared prediction error with that of a reference prediction equal to the mean observed grade in the evaluated sample. Its maximum value is 1, indicating perfect predictions. A negative value indicates greater squared prediction error than the mean-based reference. Higher values indicate better predictive performance on the same evaluation sample.

(9) $$R^{2}=1-\frac{\sum_{i=1}^{n}{\left(y_{i}-{\hat{y}}_{i}\right)}^{2}}{\sum_{i=1}^{n}{\left(y_{i}-\bar{y}\right)}^{2}}$$

where $\bar{y}$ denotes the mean observed grade in the sample used to calculate the evaluation metric.

### 2.4. Experimental Setup and Evaluation Protocol

All experiments were conducted using Python 3.9 (Python Software Foundation, Beaverton, OR, USA) on Windows 10 (Microsoft Corporation, Redmond, WA, USA). Model training was performed using an NVIDIA RTX 4060 Ti GPU (NVIDIA Corporation, Santa Clara, CA, USA).

To ensure a reliable evaluation of model generalization capability, all model training, parameter optimization, and model selection procedures were conducted exclusively using the training set. The testing set was completely isolated from the training process and was used only for the final evaluation of the developed models.

For the facial expression recognition models, including ResNet50, Vision Transformer (ViT) and the hybrid model, model parameters were optimized based on the training samples. The best-performing model on the training procedure was subsequently applied to extract emotional features from the held-out testing samples.

For academic performance prediction, regression models including random forest, XGBoost, CatBoost, ridge regression and the mixture of experts (MoE) framework were trained using the multimodal features derived from the training set. Hyperparameter configurations were determined before final testing, and no information from the testing set was involved in parameter tuning or model selection.

For the supplementary repeated evaluation, the MoE uses training set cross-fitting to construct its gating inputs. Within each outer split, the 50 training students are divided into five folds stratified by educational level. Each expert is fitted on four folds and predicts the remaining fold, producing one out-of-fold prediction per training student. Missing value imputation, numerical scaling and categorical encoding are fitted separately within the training portion of each expert fit. Consequently, the gating network receives expert predictions generated without fitting those experts to the corresponding students.

The gating network combines the four out-of-fold expert predictions with the predictors available under the evaluated input configuration. Its feature preprocessing is fitted using the 50 outer-training students only. The target grades and expert predictions are standardized using the mean and standard deviation of the outer-training grades. The network contains two fully connected hidden layers with 32 and 16 units, respectively. Each hidden layer uses ReLU activation and is followed by dropout at a rate of 0.200. Four output logits are converted into non-negative expert weights using a Softmax temperature of 6. The weights sum to one for each student.

The gating loss combines mean squared prediction error on the standardized grade scale with a KL divergence penalty between the expert weight distribution and a uniform distribution over the four experts. The KL coefficient is 0.050. The gating network is trained for 200 epochs using Adam with a learning rate of 0.001 and a batch size of 16. The experts are not jointly updated during gating optimization. The training procedure uses fixed epochs and does not use outer-test outcomes for early stopping or parameter selection.

For outer-test prediction, each expert is fitted using all 50 outer-training students. Its predictions for the 21 test students are combined by the trained gating network, using preprocessing parameters estimated from the outer-training data. Final MoE predictions are calculated as weighted averages of the expert predictions on the original grade scale. This separation concerns the downstream experts and gating network; it does not independently verify the training provenance of the available upstream representations.

Expert weight analysis focused on configuration D with ϖ = 2 and k = 20. Only weights for outer-test students were included. For each student, the weight assigned to each expert was first averaged across the splits in which that student appeared in the test set. These student-level averages were then summarized separately for the 43 undergraduate and 28 postgraduate students using means and sample standard deviations. The reported standard deviations therefore describe variation between students. Educational level was used for descriptive grouping rather than as a gating predictor. Group differences were interpreted descriptively, without claims of statistical significance.

Facial state recognition was evaluated on 4625 images from the 21 test students in the designated split with seed 42. ResNet50, ViT and the hybrid model were evaluated using the same images and reference labels. Overall accuracy was calculated as the proportion of correctly classified images. For class-specific evaluation, each category was treated as the positive class in turn. Precision, recall and F1 were calculated as TP/(TP + FP), TP/(TP + FN) and 2TP/(2TP + FP + FN), respectively. Here, TP, FP and FN denote true-positive, false-positive and false-negative counts for the class being evaluated. Macro-averaged F1 was the arithmetic mean of the two class-specific F1 scores. Confusion matrices recorded the counts for each combination of reference and predicted labels.

Agreement on the independently labeled 600-image subset was assessed using percentage agreement and Cohen’s κ, calculated as

(10) $$\kappa =\frac{p_{o}-p_{e}}{1-p_{e}}$$

Here, $p_{o}$ denotes observed agreement, and $p_{e}$ denotes agreement expected from the annotators’ marginal label proportions. Confidence intervals for accuracy and annotation agreement were estimated using 5000 student cluster bootstrap resamples. Each resample retained all evaluated images belonging to the sampled students, accounting for the clustering of images within students. These intervals describe uncertainty conditional on the evaluated images, labels and predictions and do not include variability from model retraining.

After completing all training procedures, the final models were evaluated on the independent testing set to obtain the reported performance metrics, including R^2^, MAE and MSE.

#### 2.4.1. Repeated Student-Level Evaluation and Baseline Comparisons

To assess sensitivity to sample allocation in this small cohort, downstream prediction was evaluated using 30 repeated student-level splits with random seeds 42 and 1–29. Each split contained 50 training students and 21 test students. Undergraduate students contributed 30 training and 13 test observations, whereas postgraduate students contributed 20 training and 8 test observations. Identical partitions were used across models and input configurations. Performance was summarized as the mean and sample standard deviation of R^2^, MAE and MSE across the 30 splits.

Three input configurations were compared: P, prerequisite course performance alone; A, prerequisite course performance together with structured educational information; and D, configuration A supplemented with ZHCJ. The D—P comparison measures the combined increment over prerequisite course performance, whereas D—A evaluates the increment associated with adding ZHCJ.

Two additional configurations were included to examine the contribution of the affective representation. Configuration B added three summary statistics to configuration A: the mean and standard deviation of the three stage scores, and the late-stage score minus the early-stage score. The standard deviation was calculated using a divisor of three. Configuration C added the early-, middle- and late-stage scores directly as three separate predictors. Configuration D instead added ZHCJ to configuration A. The structured inputs and prerequisite course information were identical across B, C and D, and the configurations used the same student-level partitions. Comparisons of D with B and C were conducted separately for XGBoost and MoE.

For the designated reference split (seed 42), 95% confidence intervals were estimated using 5000 student-level bootstrap resamples, stratified by educational level. These intervals describe the uncertainty conditional on the reference test set and its predictions, rather than uncertainty in the mean across the 30 splits. The repeated holdout design was used to examine sensitivity to sample allocation while preserving the training and test sizes of the initial evaluation. Using matched partitions also enabled comparisons between models and input configurations under the same evaluation conditions. This analysis assesses downstream prediction using the available student-level representation. It does not, by itself, establish the stability of the upstream representation learning process or exclude overfitting at that stage.

Descriptive subgroup evaluation was conducted for the MoE model under configuration D using the gender and political affiliation categories recorded in the dataset. MAE was calculated separately for each subgroup within each test split and summarized as the mean ± sample standard deviation across splits containing at least one test student from that subgroup. Subgroup sizes refer to the full cohort, whereas the number of test students varied between splits. The analysis focused on MAE because group-specific R^2^ can be unstable or undefined when subgroup samples are small or exhibit limited grade variation. Ethnicity was constant within the cohort and was retained in the predictor set; no ethnicity-based subgroup evaluation was conducted. No tests of equivalence or subgroup performance differences were conducted.

Early-, middle- and late-stage scores were visualized separately for undergraduate and postgraduate students. The descriptive analysis included 71 students with complete three-stage records: 43 undergraduates and 28 postgraduates. Group means and pointwise 95% confidence intervals were estimated using 10,000 student-level percentile bootstrap resamples within each educational group. Each resample retained a student’s three scores together.

#### 2.4.2. Statistical Comparisons

Model comparisons used paired differences calculated on identical student-level splits. For each metric, the difference between MoE and XGBoost under configuration D was defined as the MoE value minus the XGBoost value. Because students were reused across repeated splits, the split-level estimates were not independent. An approximate corrected resampled t-test was therefore used following Nadeau and Bengio [48]. The corrected standard error was calculated as

(11) $$SE_{\mathrm{corr}}=\sqrt{(1/30+21/50)s_{d}^{2}}$$

where $s_{d}^{2}$ denotes the sample variance of the 30 paired differences. Two-sided tests and pointwise 95% confidence intervals were calculated using a t distribution with 29 degrees of freedom.

Holm adjustment [49] was applied jointly to the 78 tests in the main repeated-split comparison set, comprising 26 contrasts evaluated using R^2^, MAE and MSE. Statistical significance was assessed at an adjusted *p*-value below 0.050. The confidence intervals were not adjusted for multiple comparisons. These analyses concern downstream prediction within the available cohort and do not quantify uncertainty arising from the complete representation learning pipeline.

#### 2.4.3. Sensitivity Analysis of the Scoring Parameters

The main analysis used ϖ = 2 and k = 20. Sensitivity to these choices was examined using nine combinations of ϖ ∈ {1, 2, 3} and k ∈ {10, 20, 40}. This grid included equal weighting of key and non-key instructional periods, as well as weaker and stronger prior smoothing. Stage scores were recalculated for each combination. The corresponding student-level representations were evaluated using the same 30 student-level partitions and structured inputs.

Differences from the main analysis setting were calculated within matched splits. The corrected resampled t procedure described in Section 2.4.2 was used, with Holm adjustment applied jointly to the 120 sensitivity tests: eight alternative settings, five regression models and three evaluation metrics. These tests formed a separate comparison family from the 78 main analysis tests. The sensitivity analysis concerns downstream prediction conditional on the available parameter-specific representations and does not establish the stability of the complete representation learning procedure. Its purpose was to characterize variation over the examined grid rather than to establish a globally optimal parameter combination.

## 3. Results

### 3.1. Facial State Classification Performance

**Figure 4:**
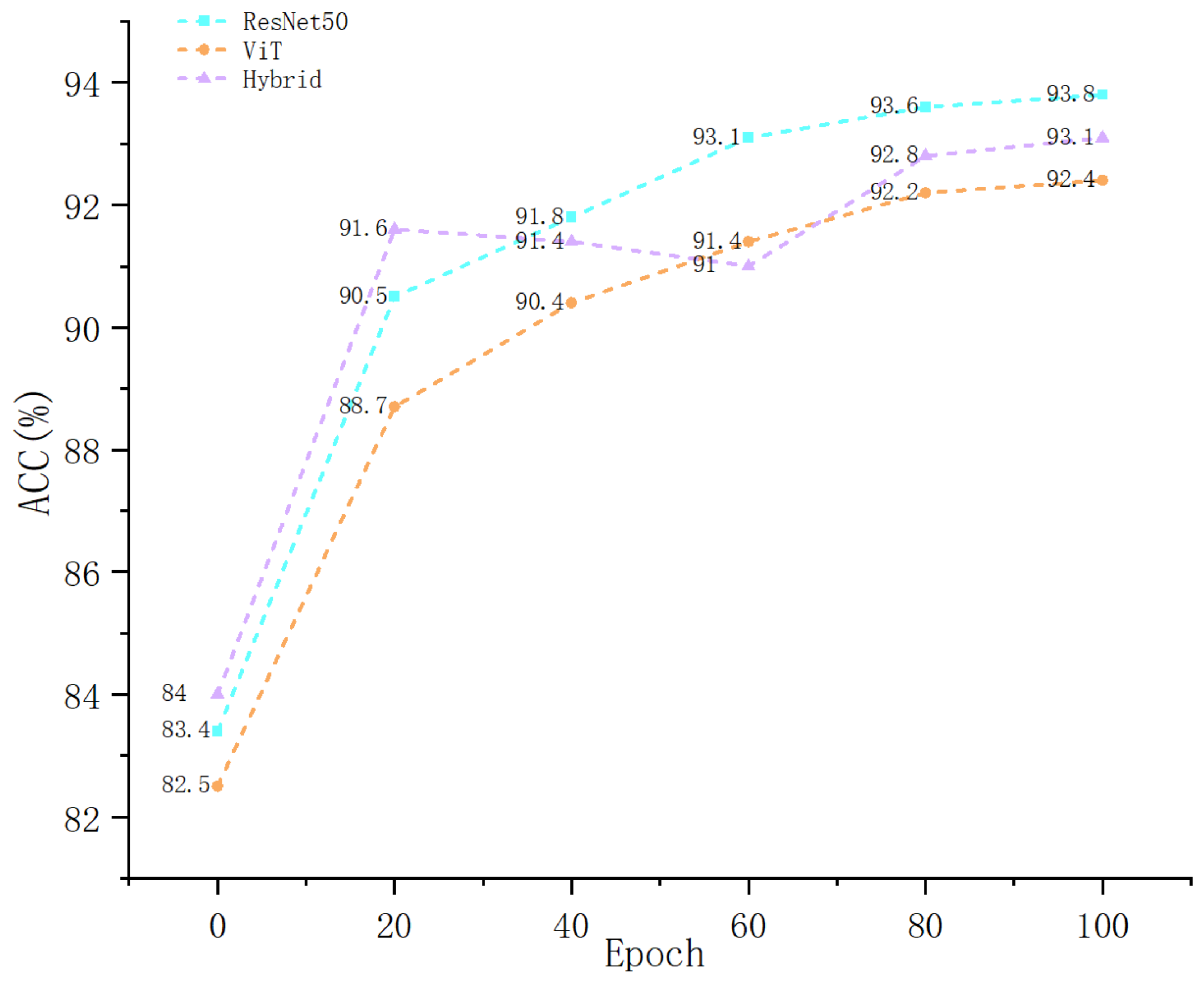
Accuracy of the three facial state classifiers across training epochs.

Three models—ResNet50, Vision Transformer (ViT) and a hybrid model—were compared on the FaceImage dataset for positive–negative facial state recognition. After 100 training epochs, ResNet50 achieved the highest observed classification accuracy of 93.8%, followed by the hybrid model at 93.1% and ViT at 92.4%. The training curves and model configurations are presented in Figure 4 and Table 4, respectively.

These results compare the three classifiers under their respective training configurations. Because the optimizers and other training settings differed, the observed accuracy differences cannot be attributed solely to network architecture. ResNet50 was adopted for subsequent stage score construction because it achieved the highest observed accuracy among the evaluated configurations.

Class-specific performance and confusion counts are presented in Table 5. ResNet50 achieved F1 scores of 0.949 for the positive category and 0.920 for the negative category, with a macro-averaged F1 of 0.935. The corresponding macro-averaged F1 scores were 0.920 for ViT and 0.927 for the hybrid model. For all three models, precision was lower for the negative category, whereas recall was more similar across categories. These differences show that overall accuracy alone does not fully describe classification performance.

The 95% student cluster bootstrap confidence intervals for accuracy were 93.104–94.474% for ResNet50, 91.684–93.176% for ViT and 92.297–93.883% for the hybrid model. These intervals describe the uncertainty conditional on the evaluated images and predictions. They exclude variability from model retraining and do not establish statistically significant differences between models. Because stage scores were calculated from predicted states, classification errors may affect subsequent analyses. These scores should therefore be interpreted as summaries of classified visual observations rather than direct measurements of students’ emotional states.

On the independently labeled subset, the two annotators agreed on 572 of 600 images, yielding an agreement rate of 95.333% (95% CI: 93.636–96.846%). Cohen’s κ was 0.903 (95% CI: 0.865–0.935). Both annotators assigned negative labels to 229 images and positive labels to 343 images, while the remaining 28 images received different labels. These results describe agreement on the operational binary categories and should not be interpreted as evidence of their psychological validity.

### 3.2. Structured Information Baselines

To establish a baseline for evaluating the incremental value of affective features and prerequisite course performance, this study trained four regression models using structured educational information alone. The predictors included students’ demographic attributes and recorded academic information, without affective features or temporal modeling. CatBoost, random forest (RF), XGBoost and ridge regression were compared for final course grade prediction. Their performance is summarized in Table 6.

XGBoost achieved the best performance among the four baseline models, with an MAE of 0.950 points, an MSE of 1.670 points^2^ and an R^2^ of 0.840. RF and ridge regression both achieved an R^2^ of 0.810, whereas CatBoost yielded an R^2^ of 0.770. These results show that structured educational information alone provided predictive value within this cohort. They also establish a reference for assessing whether affective representations and prerequisite course performance provide additional predictive information in the subsequent experiments.

### 3.3. Effects of Affective Representations and Prerequisite Course Performance

**Figure 5:**
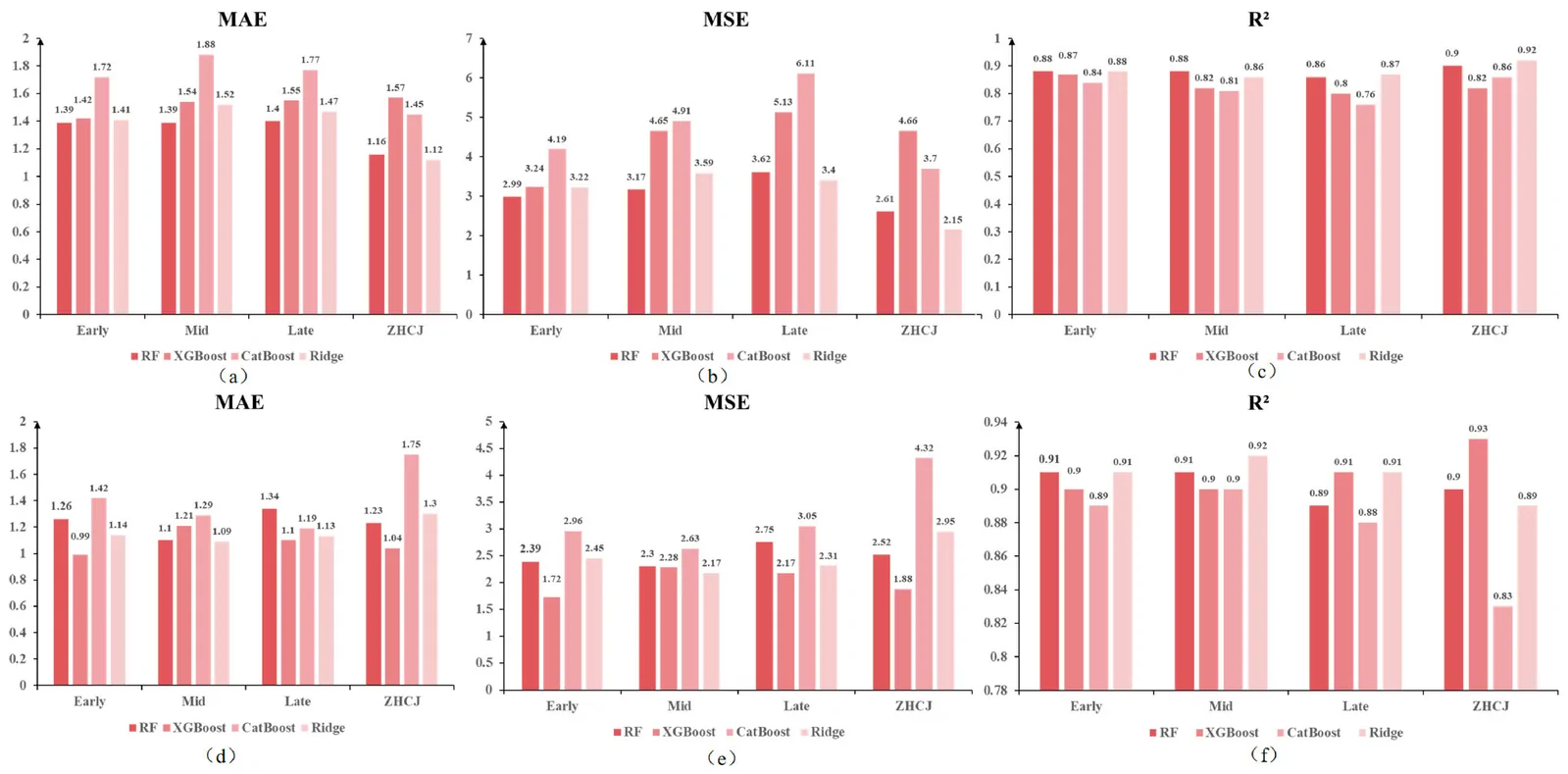
Prediction performance of four regression models: (**a**–**c**) without prerequisite course information; (**d**–**f**) with prerequisite course information. Within each group, panels show MAE, MSE and R^2^, respectively.

This study evaluated eight input configurations to examine the contributions of affective representations and prerequisite course performance. Each configuration combined structured educational information with an early-, middle- or late-stage score, or ZHCJ. The four representations were evaluated with and without the fused prerequisite course performance feature using RF, XGBoost, CatBoost and ridge regression. Figure 5 and Table 7 summarize the results.

The initial ablation results varied across regression models and affective representations (Table 7). Without prerequisite course information, ridge regression using ZHCJ achieved an MAE of 1.120 points, an MSE of 2.150 points^2^ and an R^2^ of 0.920. However, ZHCJ did not outperform every single-stage representation for every regression model. For example, without prerequisite course information, XGBoost using the early-stage score achieved a lower MAE than XGBoost using ZHCJ, at 1.420 and 1.570 points, respectively.

Adding prerequisite course information improved prediction in several configurations, but the improvement was not uniform. For XGBoost using the early-stage score, MAE decreased from 1.420 to 0.990 points and R^2^ increased from 0.870 to 0.900. In contrast, for CatBoost using ZHCJ, MAE increased from 1.450 to 1.750 points and R^2^ decreased from 0.860 to 0.830. Ridge regression using ZHCJ also showed deterioration, with MAE increasing from 1.120 to 1.300 points and R^2^ decreasing from 0.920 to 0.890.

The contribution of prerequisite course information depended on the regression model and affective representation, with no consistent improvement across all settings. These differences describe the initial held-out evaluation; repeated-split statistical comparisons are reported in Section 3.5. Previous research has examined how variability in past performance relates to students’ self-prediction accuracy [50] and compared course grades with GPA for predicting graduation performance [51].

### 3.4. MoE Prediction Performance and Expert Weights

**Figure 6:**
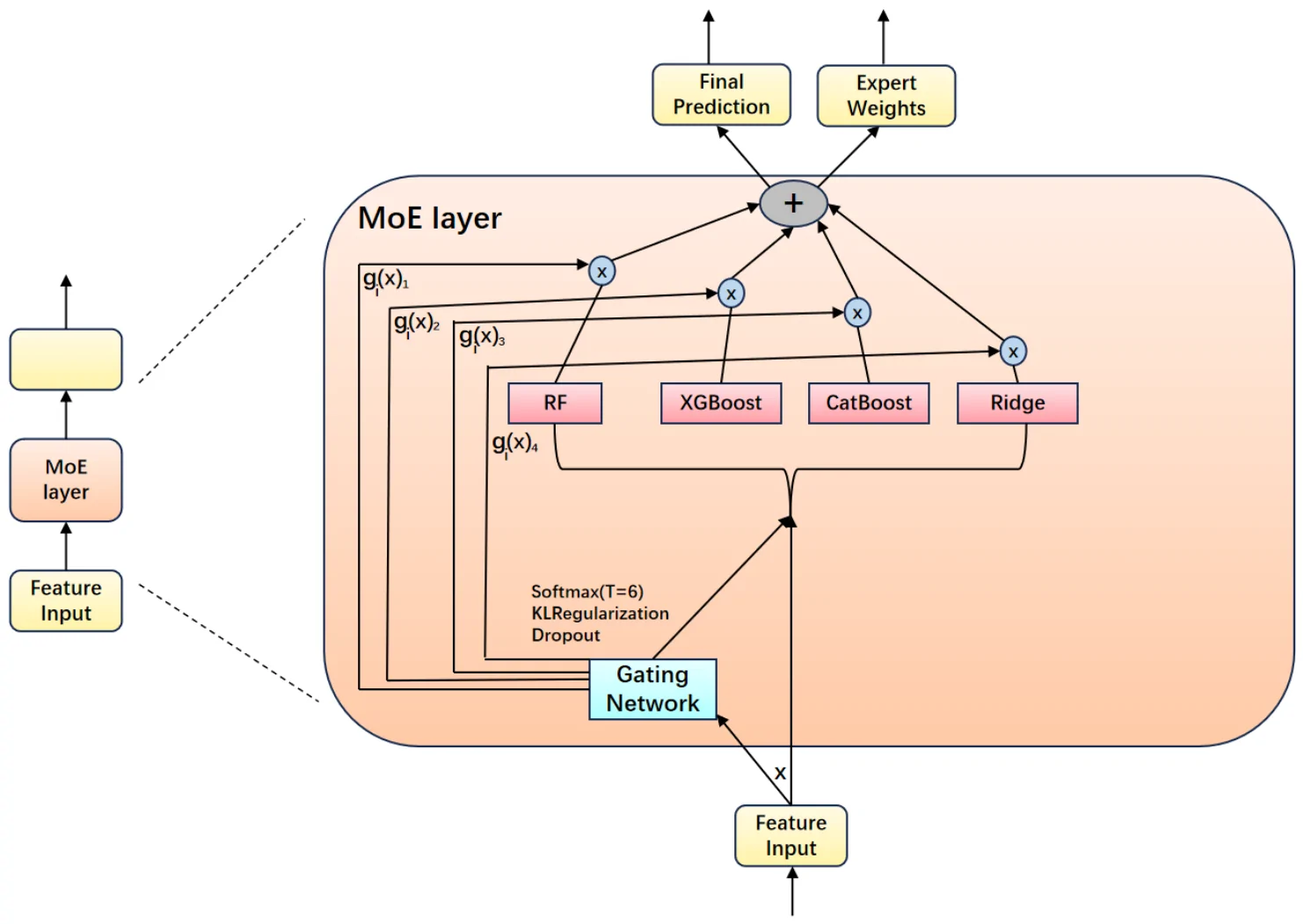
Mixture of experts (MoE) framework for student performance prediction.

Building on the ablation settings in Section 3.3, this experiment evaluates a mixture of experts (MoE) model combining random forest, XGBoost, CatBoost and ridge regression. The gating network assigns input-dependent weights to the four expert predictions. The training procedure for the supplementary repeated evaluation is described in Section 2.4, and the framework is illustrated in Figure 6. Temperature scaling, KL divergence regularization and dropout are components of the model design; their individual effects are not established by the training–test performance gap alone.

For each student, the gating network produces four non-negative weights that sum to one. The final prediction is the weighted combination of the four expert predictions. During gating training, the expert prediction inputs are out-of-fold predictions from the outer-training students; during testing, they are predictions from experts fitted on the complete outer-training set. The weights describe the allocation among experts for a given input and should not be interpreted as causal effects of student characteristics.

The basic form of the MoE model can be expressed as:

(12) $$\hat{y}=\sum_{i=1}^{M}g_{i}(x)f_{i}(x),\;\mathrm{where}\;\sum_{i=1}^{M}g_{i}(x)=1$$

Here, $f_{i}(x)$ denotes the prediction of expert i and $g_{i}(x)$ its gating weight, with M=4. The gating input comprises the available predictors and the four expert predictions. The expert outputs are on the original grade scale. Because the weights are non-negative and sum to one, the final prediction is a convex combination of the expert outputs.

**Figure 7:**
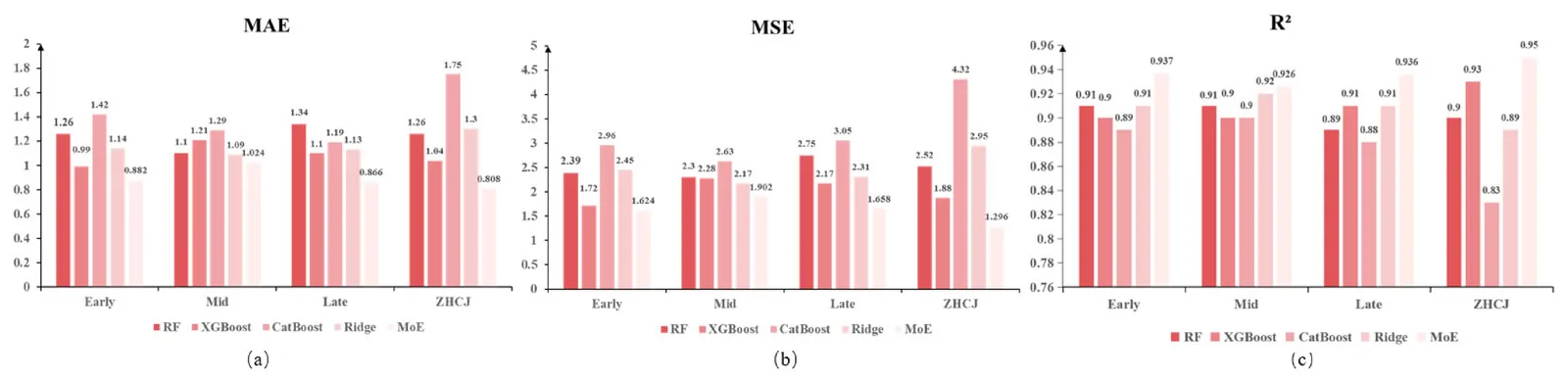
Prediction performance of random forest, XGBoost, CatBoost, ridge regression and MoE using early, mid, late and ZHCJ features: (**a**) MAE; (**b**) MSE; (**c**) R^2^.

The early-, middle- and late-stage scores and ZHCJ were evaluated separately, with the remaining inputs held constant. In the initial held-out evaluation, MoE achieved test set R^2^ values of 0.937, 0.926, 0.936 and 0.950, respectively (Figure 7 and Table 8). ZHCJ also yielded the lowest MAE and MSE, at 0.808 points and 1.296 points^2^. These single-partition results do not establish general superiority over individual regressors or robustness to distribution shifts. Repeated model comparisons are reported in Section 3.5.

In the repeated evaluation under configuration D, RF had the highest mean weight in both educational groups. For the 43 undergraduates, mean weights ± between-student standard deviations were 0.397 ± 0.064 for RF, 0.244 ± 0.197 for XGBoost, 0.142 ± 0.137 for CatBoost and 0.217 ± 0.188 for ridge regression. The corresponding values for the 28 postgraduates were 0.454 ± 0.035, 0.248 ± 0.194, 0.197 ± 0.150 and 0.101 ± 0.093.

Postgraduates had higher mean RF and CatBoost weights, whereas undergraduates had a higher mean ridge weight; mean XGBoost weights were similar. These allocations describe how the model combines its experts rather than the importance of individual input features. A higher weight does not establish that an expert is the most accurate stand-alone predictor, and group differences do not identify causal effects of educational level or other student characteristics.

Input-dependent routing has been studied in sparse MoE architectures [52], while Ma et al. developed multi-gate MoE for modeling relationships among multiple tasks [53]. Multi-model fusion has also been applied to academic performance prediction [54]. These studies provide methodological context; the present model combines four regression experts for a single prediction task.

### 3.5. Repeated Student-Level Evaluation and Affective Representation Comparisons

To complement the initial held-out comparisons, MoE and XGBoost were evaluated using five input configurations (P, A, B, C and D) across 30 matched student-level splits. Each split included 50 training students and 21 test students, with identical partitions used across models and configurations. Table 9 defines the configurations and summarizes their predictive performance.

Under configuration D, MoE achieved a mean R^2^ of 0.943 ± 0.002 and an MAE of 1.144 ± 0.128 points (Table 9). Compared with configuration P, mean R^2^ increased by 0.150 (95% CI: 0.121–0.179), while mean MAE decreased by 1.036 points (difference: −1.036; 95% CI: −1.279 to −0.793). Relative to configuration A, adding ZHCJ increased mean R^2^ by 0.091 (95% CI: 0.072–0.110) and reduced mean MAE by 0.694 points (difference: −0.694; 95% CI: −0.865 to −0.523). All four comparisons remained significant after Holm adjustment across the 78 main analysis tests (*p* < 0.001). The D—P comparison reflects the combined contribution of structured educational information and ZHCJ, whereas D—A reflects the increment associated with ZHCJ.

For the reference split (seed 42), MoE under configuration D achieved an R^2^ of 0.938 (95% CI: 0.931–0.942). Its MAE was 1.348 points (95% CI: 1.003–1.754). These intervals were estimated from 5000 student-level bootstrap resamples stratified by educational level. They describe uncertainty conditional on the reference test set and its predictions, rather than confidence intervals for the mean across the 30 splits.

Under configuration D, MoE achieved a mean R^2^ 0.015 higher and a mean MAE 0.130 points lower than XGBoost (Table 10). Both differences remained significant after Holm adjustment (*p* < 0.001). These findings support an additional benefit of expert aggregation under the evaluated configuration, alongside the incremental predictive value of ZHCJ. Repeated splits assess sensitivity to student allocation within the same cohort; they do not increase the number of independent participants or establish performance in external populations.

For MoE under configuration D, MAE was 1.113 ± 0.121 points for female students (*n* = 63) and 1.385 ± 0.893 points for male students (*n* = 8). By political affiliation, MAE was 1.185 ± 0.256 points for Communist Party of China members (*n* = 7), 1.107 ± 0.163 points for Communist Youth League members (*n* = 52) and 1.275 ± 0.212 points for non-affiliated students (*n* = 12). Group sizes refer to the full cohort. Values are means ± sample standard deviations across test splits containing each subgroup. Female students, Youth League members and non-affiliated students appeared in all 30 test splits; male students and Communist Party members appeared in 28 and 29 splits, respectively. These descriptive results do not establish equivalent prediction performance across groups.

Among configurations B, C and D, configuration D achieved the highest mean R^2^ and the lowest mean MAE and MSE for both predictors (Table 9). MoE achieved mean R^2^ values of 0.898, 0.909 and 0.943 under B, C and D, respectively. The corresponding mean MAE values were 1.525, 1.446 and 1.144 points. XGBoost showed the same ordering.

Paired comparisons supported the advantage of configuration D over both simpler representations (Table 10). For MoE, the mean R^2^ differences for D—B and D—C were 0.044 and 0.034, respectively, with mean MAE reductions of 0.381 and 0.302 points. For XGBoost, the corresponding R^2^ differences were 0.056 and 0.037, with MAE reductions of 0.430 and 0.299 points. All eight representation comparisons remained significant after Holm adjustment across the 78 main analysis tests (*p* < 0.001).

Together, these results support the predictive advantage of the Bi-LSTM-derived representation over the evaluated summary statistics and directly entered stage scores. The consistent direction of improvement across MoE and XGBoost indicates that this advantage was not confined to a single downstream predictor. However, the comparison concerns three-stage observations and the specified alternatives; it does not establish that Bi-LSTM is indispensable or superior to all other sequence models.

**Figure 8:**
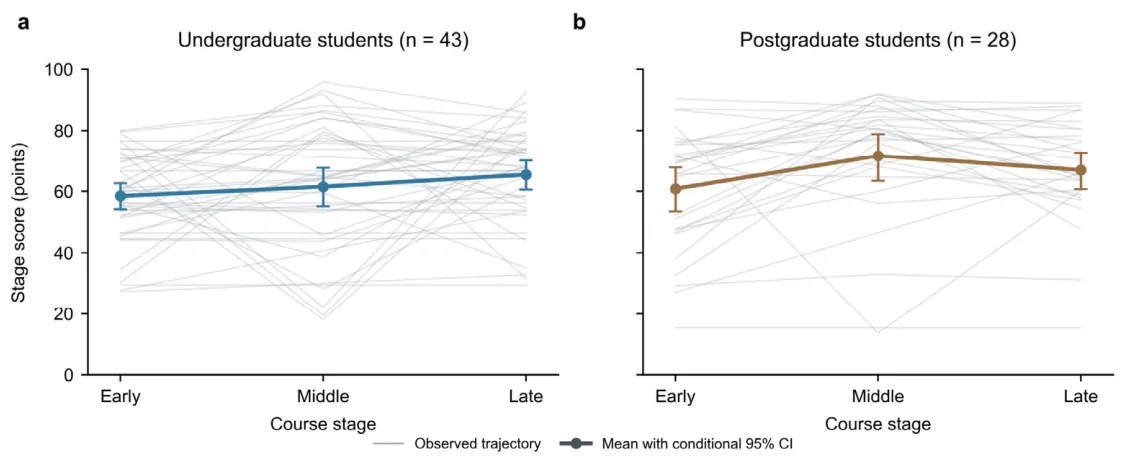
Three-stage score trajectories: (**a**) undergraduate students (*n* = 43); (**b**) postgraduate students (*n* = 28). Gray lines show individual trajectories. Colored lines and markers represent group means, and error bars indicate pointwise 95% bootstrap confidence intervals.

Figure 8 shows the three-stage score trajectories of undergraduate and postgraduate students. Undergraduate mean scores increased from 58.464 points in the early stage to 61.490 in the middle stage and 65.438 in the late stage. Postgraduate means were 60.822, 71.636 and 66.947 points, respectively, with the highest mean score in the middle stage.

### 3.6. Sensitivity to the Scoring Parameters

The main analysis used ϖ = 2 and k = 20. Sensitivity was assessed using nine combinations of ϖ ∈ {1, 2, 3} and k ∈ {10, 20, 40}, with the same 30 student-level splits used across settings. Table 11 summarizes the results for MoE and XGBoost. Within the examined grid, the main analysis combination achieved the highest mean R^2^ and the lowest mean MAE and MSE for both models.

Across the examined combinations, mean R^2^ ranged from 0.933 to 0.943 for MoE and from 0.915 to 0.927 for XGBoost. The corresponding mean MAE ranged from 1.144 to 1.234 points and from 1.274 to 1.376 points, respectively. The combination ϖ = 1 and k = 40 produced the lowest mean R^2^ and the highest mean MAE and MSE for both models.

Compared with each of the eight alternative settings, the main analysis combination achieved significantly higher R^2^ and lower MAE for both models after Holm adjustment across the 120 sensitivity tests (all adjusted *p* < 0.001). MSE was also significantly lower in all corresponding comparisons (all adjusted *p* < 0.005). Thus, all 48 comparisons involving MoE and XGBoost favored the main analysis combination after multiplicity adjustment.

The closest alternative in mean performance was ϖ = 3 and k = 20. For MoE, the main setting yielded a mean R^2^ 0.001 higher (95% CI: 0.001–0.002) and a mean MAE 0.013 points lower (difference: −0.013; 95% CI: −0.017 to −0.010). For XGBoost, the corresponding differences were 0.002 in R^2^ (95% CI: 0.001–0.002) and −0.014 points in MAE (95% CI: −0.019 to −0.010). These pointwise 95% confidence intervals were estimated using the corrected resampled t procedure and were not adjusted for multiplicity.

These results identify ϖ = 2 and k = 20 as the best-performing combination among the nine settings evaluated in this cohort. The gains over the nearest alternative were small in absolute terms, so statistical significance alone does not establish educational importance. Optimality outside the examined grid or in other cohorts remains unestablished.

## 4. Discussion

In online classroom environments, emotion modeling should not be confined to facial expression variations in the visual channel, but should also account for non-visual signals—such as social semantic cues—that influence learners’ cognitive and affective states. A study by Huang et al. (2025), based on data from online learning forums, demonstrated that perceived social support during the learning process can significantly alleviate students’ learning burnout, with this effect being moderated by learners’ levels of autonomy [55]. This finding suggests that educational evaluation systems designed for online teaching contexts should explicitly consider the interaction mechanisms among multi-channel emotional and cognitive information, thereby enhancing both model interpretability and the practical value of educational interventions. Although the present study focuses on the temporal modeling of visual facial expressions, future work may integrate linguistic–semantic and social interaction features to further improve the completeness of multimodal assessment systems.

The predictive contribution of ZHCJ should be assessed against alternative representations derived from the same three stages. The initial held-out comparisons reported variation across regression models and input configurations, whereas the repeated student-level evaluation provided matched comparisons with summary statistics and directly entered stage scores (Table 9 and Table 10). ZHCJ achieved higher mean R^2^ and lower mean MAE than both alternatives under MoE and XGBoost. Relative to directly entered stage scores, ZHCJ increased mean R^2^ by 0.034 for MoE and 0.037 for XGBoost, while reducing mean MAE by 0.302 and 0.299 points, respectively. All four differences remained significant after Holm adjustment across the main comparison set (*p* < 0.001). The consistent improvement across these two downstream predictors supports the predictive value of the evaluated representation within this cohort.

These comparisons provide stronger support for the adopted Bi-LSTM-derived representation than comparisons with individual stage scores alone, because the alternatives also incorporate observations from all three stages. Nevertheless, the sequences contain only three time steps. The findings therefore concern the integration of early-, middle- and late-stage information and do not establish the learning of long-range dependencies. They also do not demonstrate that Bi-LSTM is indispensable or superior to every alternative sequence architecture. Likewise, the initial performance of ridge regression does not establish greater stability or resistance to multicollinearity, as these properties were not directly evaluated. The initial held-out results and the repeated comparisons should be interpreted according to their respective input configurations and evaluation procedures.

Prerequisite course performance was included as an indicator of prior academic achievement and aggregated using the knowledge state mapping approach described in Section 2.3.3. Its contribution was not uniform across the initial ablation settings. Although XGBoost improved in several configurations, CatBoost and ridge regression showed poorer performance when prerequisite course information was added to ZHCJ (Table 7). Consequently, these results should not be interpreted as evidence that adding prerequisite course information necessarily improves prediction. They instead indicate that the utility of this information depends on the model and its combination with other predictors. The initial held-out results and the repeated student-level evaluation should also be interpreted separately because they summarize different evaluation procedures.

Input-dependent expert routing has been explored in sparse MoE architectures [52]. In the present framework, four regression experts are combined using student-specific weights. The repeated student-level comparison showed that MoE outperformed XGBoost under configuration D, with a mean R^2^ difference of 0.015 (95% CI: 0.012–0.018) and an MAE difference of −0.130 points (95% CI: −0.163 to −0.098). Both differences remained significant after Holm adjustment across the main comparison set (*p* < 0.001). These results support an additional predictive benefit of expert aggregation within the evaluated cohort. This benefit should be distinguished from the contribution of the affective representation, which was assessed through comparisons between input configurations. The MoE–XGBoost comparison evaluates the combined architecture and does not isolate the individual effects of temperature scaling, KL regularization or dropout.

The sensitivity analysis further showed that the main analysis setting, ϖ = 2 and k = 20, achieved the best mean performance among the nine examined parameter combinations. For both MoE and XGBoost, its advantages over the eight alternatives remained significant after adjustment across the 120 sensitivity tests. However, the differences from the nearest alternative, ϖ = 3 and k = 20, were small in absolute terms. The findings therefore support this setting within the evaluated grid without establishing that the numerical gains are educationally important or that the combination is optimal outside the examined range.

Table 12 compares the present framework with selected academic performance prediction studies in terms of sample size, data sources, modeling approach and reported performance. These studies represent clickstream-based sequence modeling, multimodal learning analytics and regression using structured student information.

Repeated splits of the same 71 students assess sensitivity to sample allocation but do not increase the number of independent participants or constitute external validation. All test students belonged to the same study cohort, and performance across other courses, institutions and student populations remains to be established. The limited number of independent student trajectories is particularly relevant to interpreting the Bi-LSTM representation: multiple facial images provide additional observations within students but do not replace independent student sequences. The observed predictive advantages consequently do not exclude overfitting during representation learning or establish the adequacy of the sample size for broader generalization.

The subgroup analysis also requires cautious interpretation. The cohort was imbalanced by gender and political affiliation, and some test splits contained very few students from the smaller groups. The larger variation in male student MAE illustrates the sensitivity of these summaries to sample allocation. Group differences may also reflect variation in grades and other characteristics. The analysis therefore provides a descriptive check of prediction errors, while fairness assessment requires larger and more balanced samples and an explicitly defined evaluation criterion.

Classroom applications of camera-based affective sensing require attention to student privacy, autonomy and the possible psychological pressure associated with being observed. Within a human-centered AI perspective, predictive performance should be evaluated alongside students’ understanding and acceptance of the system. The positive–negative categories provide operational descriptions of observable classroom states; their interpretation should account for the instructional context and individual differences. Model outputs should support dialog between teachers and students and should not serve as the sole basis for grading, disciplinary action or judgments about student effort.

Future deployment should clearly explain what information is recorded, how derived scores are used, who can access the data and how long records are retained. Data collection and access should be limited to the stated educational purpose, with identifiable images restricted to authorized personnel. Students should have meaningful opportunities to decline research recording without academic disadvantage and to question or correct interpretations of their classroom states. Prospective evaluation should assess perceived monitoring pressure, acceptance and effects on classroom participation alongside predictive performance. Such evaluation would help determine whether the framework can provide useful educational support while respecting students’ autonomy and maintaining a supportive learning environment.

Overall, this study supports incorporating stage-level classroom affective information into academic performance prediction. A systematic review has reported relationships between academic emotions and learning outcomes that vary with emotional states and learning contexts [56]. Previous research has also applied attention-based LSTM models to student performance prediction [38]. In the present study, ZHCJ outperformed the evaluated summary statistics and directly entered stage scores under both MoE and XGBoost. Expert aggregation provided an additional predictive benefit under configuration D, while the contribution of prerequisite course information depended on the model and input configuration. These findings support the predictive value of the evaluated framework within this cohort and motivate further evaluation of its use in educational support.

## 5. Conclusions

This study proposes Bi-LSTM-SCPM, a student performance prediction framework that combines classroom affective information with structured educational records and prerequisite course performance. ResNet50 classifies sampled facial images into positive and negative classroom states. Teacher-identified key instructional periods and Bayesian smoothing are then used to construct early-, middle- and late-stage scores. Bi-LSTM integrates these three stage scores into the student-level affective representation ZHCJ, which is combined with the structured inputs for final grade prediction. The temporal component therefore concerns the integration of observations across three course stages. Prediction using the complete representation is performed after the late-stage observations become available.

The repeated student-level evaluation supported the incremental predictive value of ZHCJ when added to structured educational information and prerequisite course performance. Across 30 matched student-level splits, MoE achieved an R^2^ of 0.943 ± 0.002 and an MAE of 1.144 ± 0.128 points under configuration D, compared with 0.927 ± 0.002 and 1.274 ± 0.149 points for XGBoost. MoE achieved significantly higher R^2^ and lower MAE after Holm adjustment (*p* < 0.001 for both metrics), supporting an additional benefit of expert aggregation. ZHCJ also achieved higher R^2^ and lower MAE than summary statistics and directly entered stage scores under both predictors. Within the nine-setting sensitivity grid, ϖ = 2 and k = 20 achieved the best mean performance and significantly outperformed the eight alternatives for both models after Holm adjustment. These findings support the predictive value of the affective representation and expert aggregation within the evaluated cohort, while validation across independent courses, institutions and student populations remains necessary.

Several limitations should be considered. First, the cohort comprised only 71 independent students, which limits the assessment of model stability and generalization. Repeated student-level splits quantify sensitivity to sample allocation but cannot exclude overfitting during representation learning or establish transferability beyond the course studied. Second, the identification of instructional key segments relies on manual annotation, which limits scalability. Third, the framework primarily uses visual affective cues and does not incorporate classroom speech or other interactional signals. The fixed 20 s sampling interval may also miss brief facial events, and its sensitivity to rapid affective changes was not evaluated using denser sampling. Finally, the system operates offline and has not undergone prospective evaluation during ongoing teaching.

Future work may extend this research in three directions to address the limitations above: (1) developing automatic detection mechanisms for instructional key segments to reduce manual dependency; (2) incorporating multi-source behavioral data, such as questioning frequency and response patterns, to enrich the representation of students’ learning states; and (3) implementing real-time prediction and feedback mechanisms compatible with online learning platforms. Expert weight summaries could help explain how predictions are combined, while prospective studies should assess whether these extensions improve instructional feedback and student outcomes.

## Figures and Tables

**Table 1:** Recording stages and observation focus.

Recording Stage	Course Weeks	Mode of Action
Early stage	1–4	Initial reactions and attitudes toward online courses
Middle stage	5–8	Changes in learning progress and attitudes
Late stage	9–12	Understanding of course content

**Table 2:** Definitions of the six classroom states in TeMSA.

Classroom State	Description
Focused	The student maintains eye contact with the screen, leans forward slightly and shows attentive body posture (e.g., nodding or taking notes).
Pleasure	Characterized by a relaxed facial expression with upward-curved lips, raised eyebrows and active participation in classroom interactions.
Head-down	The student frequently lowers their head, avoiding eye contact with the screen, often accompanied by distracted behavior.
Phone-using	The student holds or uses a mobile device, with their gaze shifted away from the screen.
Head-turning	The student turns their head sideways to engage in off-topic conversations or shifts attention away from teaching content.
Drowsiness	Exhibits signs of fatigue, such as frequent yawning, drooping eyelids or resting their head on their hands.

**Table 3:** Variables in the EI dataset.

Category	Columns	Features
Demographic information	4	Gender, political affiliation, ethnicity, place of origin
Academic records	3	Daily performance, final examination score, prerequisite course performance

**Note:** Final examination score was the prediction target; the remaining six variables were predictors.

**Table 4:** Model-specific training settings for the facial state classifiers.

Parameter	ResNet50	ViT	Hybrid
Optimizer	SGD with momentum	AdamW	RMSprop
Learning rate	1 × 10^−2^	1 × 10^−4^	1 × 10^−4^
Optimizer-specific coefficient	Momentum: 0.900	Weight decay: 0.010	Rho: 0.900
Classification head dropout rate	0.500	0.100	0.500

**Note:** SGD denotes stochastic gradient descent; rho is the RMSprop moving average coefficient. The reported dropout rates apply only to the classification heads.

**Table 5:** Class-specific metrics and confusion counts for the three facial state recognition models.

Model	Class	Predicted Negative	Predicted Positive	Precision	Recall	F1
ResNet50	Negative	1650	113	0.905	0.936	0.920
ResNet50	Positive	174	2688	0.960	0.939	0.949
ViT	Negative	1627	136	0.883	0.923	0.903
ViT	Positive	215	2647	0.951	0.925	0.938
Hybrid	Negative	1625	138	0.900	0.922	0.911
Hybrid	Positive	181	2681	0.951	0.937	0.944

**Note:** All models were evaluated on the same 4625 images from 21 held-out students. Prediction columns contain image counts, with reference classes shown by row. Each category was treated as the positive class when calculating its precision, recall and F1. Calculations used unrounded values. Overall accuracies derived from these counts round to 93.8%, 92.4% and 93.1% for ResNet50, ViT and the hybrid model, respectively.

**Table 6:** Performance of baseline models using only structured educational information.

Model	MAE (Points)	MSE (Points^2^)	R^2^
CatBoost	1.310	3.280	0.770
Random Forest	1.190	2.320	0.810
XGBoost	0.950	1.670	0.840
Ridge	1.130	2.320	0.810

**Table 7:** Prediction performance with and without prerequisite course information.

Index	RF1	RF2	XGBoost1	XGBoost2	CatBoost1	CatBoost2	Ridge1	Ridge2
MAE (Early)	1.39	1.26	1.42	0.99	1.72	1.42	1.41	1.14
MAE (Mid)	1.39	1.1	1.54	1.21	1.88	1.29	1.52	1.09
MAE (Late)	1.4	1.34	1.55	1.1	1.77	1.19	1.47	1.13
MAE (ZHCJ)	1.16	1.23	1.57	1.04	1.45	1.75	1.12	1.3
MSE (Early)	2.99	2.39	3.24	1.72	4.19	2.96	3.22	2.45
MSE (Mid)	3.17	2.3	4.65	2.28	4.91	2.63	3.59	2.17
MSE (Late)	3.62	2.75	5.13	2.17	6.11	3.05	3.4	2.31
MSE (ZHCJ)	2.61	2.52	4.66	1.88	3.7	4.32	2.15	2.95
R^2^ (Early)	0.88	0.91	0.87	0.9	0.84	0.89	0.88	0.91
R^2^ (Mid)	0.88	0.91	0.82	0.90	0.81	0.9	0.86	0.92
R^2^ (Late)	0.86	0.89	0.8	0.91	0.76	0.88	0.87	0.91
R^2^ (ZHCJ)	0.9	0.9	0.82	0.93	0.86	0.83	0.92	0.89

**Note:** Suffixes 1 and 2 indicate configurations without and with prerequisite course information, respectively. MAE and MSE are expressed in grade points and squared grade points, respectively; R^2^ is dimensionless.

**Table 8:** Initial held-out prediction performance of MoE using four affective representations.

Affective Representation	MAE	MSE	R^2^
Early-stage score	0.882	1.624	0.937
Middle-stage score	1.024	1.902	0.926
Late-stage score	0.866	1.658	0.936
ZHCJ	0.808	1.296	0.950

**Note:** All configurations included the same structured educational information and prerequisite course performance. Results for the individual regression models are reported in Table 7.

**Table 9:** Prediction performance across 30 matched student-level splits.

Model	Configuration	R^2^	MAE	MSE
MoE	P	0.793 ± 0.022	2.180 ± 0.290	6.310 ± 1.963
MoE	A	0.852 ± 0.014	1.837 ± 0.240	4.525 ± 1.456
MoE	B	0.898 ± 0.007	1.525 ± 0.187	3.100 ± 0.946
MoE	C	0.909 ± 0.006	1.446 ± 0.175	2.789 ± 0.845
MoE	D	0.943 ± 0.002	1.144 ± 0.128	1.738 ± 0.481
XGBoost	P	0.756 ± 0.024	2.352 ± 0.315	7.460 ± 2.408
XGBoost	A	0.825 ± 0.017	1.988 ± 0.262	5.373 ± 1.792
XGBoost	B	0.871 ± 0.010	1.704 ± 0.214	3.958 ± 1.285
XGBoost	C	0.890 ± 0.007	1.573 ± 0.194	3.360 ± 1.067
XGBoost	D	0.927 ± 0.002	1.274 ± 0.149	2.219 ± 0.672

**Note:** Values are means ± sample standard deviations across 30 matched student-level splits. P uses prerequisite course performance alone; A adds structured educational information to P. B, C and D extend A with stage score summary statistics (mean, standard deviation and late-minus-early difference), the three stage scores and ZHCJ, respectively. R^2^ is dimensionless. Paired differences were calculated using unrounded values.

**Table 10:** Paired model and affective representation comparisons across 30 matched student-level splits.

Contrast	ΔR^2^ (95% CI)	Holm-Adjusted *p*: R^2^	ΔMAE (95% CI)	Holm-Adjusted *p*: MAE
MoE (D)—XGBoost (D)	0.015 [0.012, 0.018]	<0.001	−0.130 [−0.163, −0.098]	<0.001
MoE: D—B	0.044 [0.036, 0.053]	<0.001	−0.381 [−0.475, −0.287]	<0.001
MoE: D—C	0.034 [0.027, 0.041]	<0.001	−0.302 [−0.378, −0.226]	<0.001
XGBoost: D—B	0.056 [0.046, 0.067]	<0.001	−0.430 [−0.532, −0.329]	<0.001
XGBoost: D—C	0.037 [0.030, 0.044]	<0.001	−0.299 [−0.372, −0.226]	<0.001

**Note:** Differences were calculated within matched splits using unrounded values and then averaged. Positive ΔR^2^ and negative ΔMAE favor MoE in the between-model comparison and configuration D in the representation comparisons. Confidence intervals are pointwise 95% intervals from the corrected resampled t procedure and are not adjusted for multiplicity. The *p*-values were Holm-adjusted jointly across the 78 main analysis tests. ΔMAE is expressed in grade points.

**Table 11:** Sensitivity of MoE and XGBoost prediction performance to ϖ and k across 30 matched student-level splits.

(ϖ, k)	MoE R^2^	MoE MAE	MoE MSE	XGBoost R^2^	XGBoost MAE	XGBoost MSE
(1, 10)	0.937 ± 0.002	1.197 ± 0.134	1.902 ± 0.528	0.920 ± 0.003	1.334 ± 0.156	2.432 ± 0.739
(1, 20)	0.939 ± 0.002	1.176 ± 0.131	1.838 ± 0.509	0.923 ± 0.003	1.311 ± 0.152	2.349 ± 0.711
(1, 40)	0.933 ± 0.002	1.234 ± 0.137	2.021 ± 0.563	0.915 ± 0.004	1.376 ± 0.160	2.587 ± 0.788
(2, 10)	0.941 ± 0.002	1.164 ± 0.130	1.802 ± 0.500	0.925 ± 0.003	1.297 ± 0.152	2.302 ± 0.700
**(2, 20)**	**0.943 ± 0.002**	**1.144 ± 0.128**	**1.738 ± 0.481**	**0.927 ± 0.002**	**1.274 ± 0.149**	**2.219 ± 0.672**
(2, 40)	0.938 ± 0.002	1.193 ± 0.135	1.892 ± 0.532	0.921 ± 0.003	1.330 ± 0.157	2.417 ± 0.743
(3, 10)	0.939 ± 0.002	1.181 ± 0.131	1.851 ± 0.511	0.923 ± 0.003	1.315 ± 0.153	2.364 ± 0.716
(3, 20)	0.941 ± 0.002	1.157 ± 0.129	1.778 ± 0.492	0.926 ± 0.003	1.288 ± 0.150	2.270 ± 0.689
(3, 40)	0.936 ± 0.002	1.206 ± 0.136	1.933 ± 0.541	0.919 ± 0.003	1.343 ± 0.158	2.467 ± 0.756

**Note:** Values are means ± sample standard deviations across the same 30 student-level splits. Bold indicates the main analysis setting, ϖ = 2 and k = 20, whose results match configuration D in Table 9. R^2^ is dimensionless. MAE and MSE are expressed in grade points and squared grade points, respectively. Calculations and statistical comparisons used unrounded values.

**Table 12:** Comparison with selected studies on academic performance prediction.

Study	Number of Students	Data Sources	Approach and Prediction Target	Reported Performance
Liu et al. [6]	5341	Weekly clickstream activity records	LSTM; academic outcome classification	Accuracy: 89.250%; AUC: 0.913
Chango et al. [42]	40	Learning system logs, facial emotion information and eye tracking	Ensemble with feature selection; pass–fail classification	Accuracy: 87.500%; AUC: 0.880
Doz et al. [44]	22,011	Sociodemographic and school characteristics	RF with 1000 trees; teacher-assigned mathematics-grade regression	RMSE: 0.980
Present study	71	Three-stage classroom affective information, educational records and prerequisite course performance	Bi-LSTM-derived ZHCJ with MoE; final grade regression	R^2^: 0.943 ± 0.002; MAE: 1.144 ± 0.128 points

**Note:** Results from previous studies refer to the specified configurations and retain their original outcome scales. The present study results correspond to configuration D and are means ± sample standard deviations across 30 matched student-level splits. Accuracy refers to academic outcome classification, not facial state recognition. Differences in datasets, prediction targets, scales and evaluation protocols prevent direct performance ranking across studies.

## Data Availability

The datasets generated and analyzed during the current study are not publicly available due to privacy and confidentiality considerations. For academic research purposes, anonymized derived data and supporting code may be requested from the corresponding authors, subject to institutional approval and applicable data use requirements.
